# Type-specific incidence, clearance and predictors of cervical human papillomavirus infections (HPV) among young women: a prospective study in Uganda

**DOI:** 10.1186/1750-9378-5-7

**Published:** 2010-04-09

**Authors:** Cecily Banura, Sven Sandin, Leen-Jan van Doorn, Wim Quint, Bernhard Kleter, Fred Wabwire-Mangen, Edward K Mbidde, Elisabete Weiderpass

**Affiliations:** 1 College of Health Sciences, Makerere University, Kampala Uganda; 2 Department of Medical Epidemiology and Biostatistics, Karolinska Institutet, Stockholm, Sweden; 3 DDL Diagnostic Laboratory, Voorburg, the Netherlands; 4 School of Public Health, Makerere University, Kampala, Uganda; 5 Uganda Virus Research Institute, Entebbe, Uganda; 6 Department of Etiological Research, Cancer Registry of Norway, Oslo, Norway; 7 Department of Community Medicine, University of Tromso, Tromso, Norway; 8 Department of Genetic Epidemiology, Samfundet Folkhalsan, Helsinki, Finland

## Abstract

**Background:**

While infections with human papillomavirus (HPV) are highly prevalent among sexually active young women in Uganda, information on incidence, clearance and their associated risk factors is sparse. To estimate the incidence, prevalence and determinants of HPV infections, we conducted a prospective follow-up study among 1,275 women aged 12-24 years at the time of recruitment. Women answered a questionnaire and underwent a pelvic examination at each visit to collect exfoliated cervical cells. The presence of 42 HPV types was evaluated in exfoliated cervical cells by a polymerase chain based (PCR) assay (SPF10-DEIA LiPA).

**Results:**

Three hundred and eighty (380) of 1,275 (29.8%) women were followed up for a median time of 18.5 months (inter-quartile range 9.7-26.6). Sixty-nine (69) women had incident HPV infections during 226 person-years of follow-up reflecting an incidence rate of 30.5 per 100 person-years. Incident HPV infections were marginally associated with HIV positivity (RR = 2.8, 95% CI: 0.9 - 8.3). Clearance for HPV type-specific infections was frequent ranging between 42.3% and 100.0% for high- and 50% and 100% for low-risk types. Only 31.2% of women cleared all their infections. Clearance was associated with HIV negativity (Adjusted clearance = 0.2, 95% CI: 0.1 - 0.7) but not with age at study entry, lifetime number of sexual partners and multiplicity of infections. The prevalence of low-grade squamous intraepithelial lesions (LSILs) was 53/365 (14.5%). None of the women had a high-grade cervical lesion (HSIL) or cancer. Twenty-two (22) of 150 (14.7%) HPV negative women at baseline developed incident LSIL during follow-up. The risk for LSIL appeared to be elevated among women with HPV 18-related types compared to women not infected with those types (RR = 3.5, 95% CI: 1.0 - 11.8).

**Conclusions:**

Incident HPV infections and type-specific HPV clearance were frequent among our study population of young women. These results underscore the need to vaccinate pre-adolescent girls before initiation of sexual activity.

## Background

Persistent infection with one or more high-risk HPV types is an important etiologic factor in the development of cervical cancer [1]. High-risk HPV types are extremely prevalent among young sexually active women in Uganda infecting about half of those aged 12-24 years [2]. Most infections in young women with or without cervical abnormalities are described as being transient [3-5] as approximately 90% of women cleared a specific type of HPV within 24 months [6]. Vaccination against high-risk types of HPV may be the most realistic intervention to reduce substantially the cervical cancer burden particularly in populations where Pap screening has largely been absent or ineffective [7]. Cohort studies pose challenges for researchers in Africa; particularly, retaining study subjects after recruitment. Consequently, information on type-specific HPV infections, clearance and potential risk factors is sparse. In Uganda, the only information available derives from two separate studies conducted among young primiparous [8] and young and adult women in a rural district [9]. Yet, this information is important to further the understanding of the natural history of HPV infections among Ugandan women as we prepare for the introduction of a vaccine against HPV infections. In this article we report findings on incidence, clearance and risk factors for HPV infections in a cohort of Ugandan women aged 12-24 years.

## Methods

### Study population and follow-up visits

Women were recruited and followed up between September 2002 and December 2006. A description of the study population at baseline is available elsewhere [2]. Briefly, participants were recruited among women aged 12-24 years presenting themselves for health services at Naguru Teenage Information and Health Centre (NTIHC) located in the suburbs of Kampala City. Women were eligible for participation if they were sexually active, resided within 20-kilometer radius of NTIHC and had no plans to relocate for the duration of the study. Follow-up visits were scheduled between 6-12, 13-18, and 19-24 months from baseline. Twenty-nine women who turned up for visits after 24 months were not turned away, although these visits were unscheduled.

### Gynaecological examination, collection of exfoliated cells and cytological assessment

During all visits, trained midwives explained the study aims and procedures, which included answering a questionnaire and having a pelvic examination to record visible abnormalities. In all visits after visual inspection of the vulva, a non-lubricated sterile speculum was inserted, and cervical exfoliated cells were collected. At the first visit at cohort enrolment, cervical exfoliated cells were collected with a sterile swab (Copan International) which was then placed in a labelled 15 mL holding tubes containing 5 mL of phosphate buffered saline (PBS), pH 7.2; samples were kept temporarily at 4°C for an average of 6 hours and then transferred to a freezer for storage at 20°C until shipment to the laboratory for HPV analysis. PBS is not a suitable solution for preservation of cells for liquid based cytology and consequently no liquid based cytology could be done with baseline samples. However, during follow-up, cervical exfoliated cells were collected and stored in a vial containing PreservCyt solution (Cytyc, Boxborough, MA) which is suitable both for liquid-based cytology and HPV testing. Briefly, for these follow-up samples, a broom style cytobrush (Cervex brush, Rovers Medical Devices B.V., Oss. The Netherlands) was inserted deep into the endocervical canal and rotated gently in a clockwise direction 5 times to collect cells from the endo- and ectocervix. The cytobrush containing cervical cellular material was then placed in a vial containing PreservCyt solution (Cytyc) and rinsed by pushing it to the bottom of the vial 10 times. The cytobrush was then discarded. The vials were closed and kept at room temperature until shipment in dry ice to DDL Diagnostic Laboratory, Voorburg, The Netherlands, for liquid based cytology and HPV testing. Cervical abnormalities in liquid-based cytology samples were read at the cytology department of the Slotervaart Hospital, Amsterdam, The Netherlands and classified according to the 2001 Bethesda Classification [10].

Before removal of the speculum, both for the baseline and follow up visits, visual inspection with acetic acid (VIA) and with Lugol's Iodine (VILI) was performed. A urine sample and 4 ml of blood (in heparinised tubes) were collected for pregnancy, HIV and syphilis testing, respectively. Non-pregnant women with external genital warts were treated with 2% podophylline paint. The treatment of pregnant women was deferred until after delivery. Women with vaginal discharge and cervicitis received a one-week syndromic treatment and were requested to ask their sexual partners to receive treatment.

### Isolation of HPV DNA

Total DNA was isolated from 200 μl of the suspension containing the cervical cells by the MagNa Pure LC instrument (Roche Diagnostics, Almere, The Netherlands), using the Total NA isolation kit (Roche Diagnostics, Almere, The Netherlands). DNA was eluted in 100 μl of elution buffer, and 10 μl was used for each PCR reaction. Each run contained positive and negative controls to monitor the DNA isolation, PCR and HPV detection and genotyping procedures.

### PCR testing

The short PCR fragment (SPF) 10 primer set was used to amplify a broad spectrum of HPV genotypes, as described earlier [11,12]. Briefly, this primer set amplifies a small fragment of 65 base pairs (bps) from the L1 region of HPV. Reverse primers contain a biotin label at the 5' end, which enables capture of the reverse strand onto streptavidin coated microtiter plates. Captured amplimers are denatured by alkaline treatment and a defined cocktail of digoxigenin - labeled probes, detecting a broad spectrum of HPV genotypes. This method is designated HPV DNA enzyme immunoassay (DEIA), and provides an optical density value. The same (SPF) 10 amplimers were used to identify the HPV genotype by reverse hybridisation on a line probe assay (LiPA) containing probes for 25 different genotypes [(SPF) 10 HPV LiPA version 1, Labo Bio-medical Products, Rijswijk, the Netherlands]. HPV 16, 18, 31, 33, 35, 39, 45, 51, 52, 56, 58, 59 and 68/73 were considered high-risk types and 6, 11, 34, 40, 42, 43, 44, 53, 54, 66, 70 and 74 were considered low-risk types [13]. Samples that were positive using the (SPF) 10 primer set but did not reveal any of the 25 aforementioned types, were provisionally classified as positive for HPV X and were subjected to a second round of testing, similar to the one already described, for 17 additional types including HPV 26, 30, 55, 61, 62, 64, 67, 69, 71, 82, 83, 84, 85, 87, 89, 90 and 91.

### HIV Testing

HIV-1 testing was performed at NTIHC following the National HIV Rapid Testing algorithm consisting of Determine rapid test (Abbot Diagnostics, Abbot Park, IL) as screening test, Statpak rapid test (ChemoBio Diagnostics, Systems, Inc. Medford, NY) as the confirmatory test and Unigold (Orgenics, Waltham, MA) as tie breaker [14]. For quality assurance and quality control, all HIV-positive results were confirmed by ELISA (Cambridge Bioscience, Cambridge, UK) or PCR assays (Roche Molecular Systems, Pleasanton, CA) at Makerere Medical School. Women found to be HIV-positive were referred to health institutions offering treatment, care and support services.

### Syphilis testing

Syphilis testing was performed at NTIHC using a commercially available standard Rapid Plasma Reagin (RPR) 18 mm circle card test (Quorum Diagnostics, Sacramento, CA). Samples with reactive RPR test results were referred to established laboratories for confirmation before treatment was offered to the women concerned and their partners.

### Pregnancy testing

All women were tested for pregnancy using a commercially available HCG dipstick pregnancy test (Cypress Diagnostics, Langdorpsesteenweg, Belgium), and the results were communicated immediately to each woman. By the end of the study 30 women were tested positive for pregnancy. The pregnant women were referred for free pre-natal counselling, evaluation and care within the same health care center. Only the pre-natal care services are being evaluated in our study, not the number of weeks of pregnancy or the decision if the pregnancy was or not to be kept.

### Ethical considerations

Women consented to participation in the study. The women younger than 18 years of age were treated according to the Uganda guidelines for conducting research on human beings that allows minors to assent for participation without a parent or legal guardian agreement. These guidelines are approved by the ethical review committees at the Faculty of Medicine, Makerere University, the Uganda National Council of Science and Technology, and the International Agency for Research on Cancer.

### Statistical analyses and definitions of outcomes

We analyzed incidence and clearance of HPV infection for all women who had at least one follow-up visit. Any HPV type was defined as a combined outcome if any HPV type was present. We defined HPV 16-related types as HPV 16, 31, 33, 35, 52, and 58 and HPV 18-related types as HPV 18, 39, 45, 59 and 68. Single and multiple infections were defined without considering uncharacterized HPV types (HPV X). We computed relative risk (RR) for HPV incidence and clearance using Poisson regression dividing the follow-up time in 6-month windows for risk time and events.

For incidence analysis, the risk time was measured for each specific HPV type or combined set of HPV types from the first time a woman tested negative until a positive test or the last visit.

For each of the groups of infections (low-risk, high-risk, HPV 16-related, HPV 18-related and any HPV) incidence was calculated for women free from all the infections in each group until infection of at least one of the HPV types in the group. Correspondingly clearance was calculated for all women infected with at least one of the HPV types in each group until the woman was free from infection of all the HPV types in each group. Thus, clearance will typically be a lot longer than for specific HPV types. This choice of groups of HPV types intend to measure an exposure assuming interchangeable risks within the group so a woman was not considered cleared from exposure of high-risk HPV if one high-risk infection was cleared while she was still infected with other high-risk type(s). Thus, clearance of any HPV was interpreted as being cleared from all HPV infections. Highly sexually active women as our study population would tend to have long clearance time even if each specific type is cleared fast.

Women positive for HPV X at baseline who tested positive for a known HPV type at follow-up visit were considered to have developed a new infection in the analysis of incidence but were excluded from the analysis of clearance as persistence of HPV X could not be known. For each HPV type, we fitted Poisson models including a time covariate only and models adjusting for HIV status, age at baseline (12-17, 18-20, 21-24 year), positivity for genital warts or syphilis (yes/no), and lifetime number of sexual partners (1, 2, 3, ≥4).

We evaluated prevalent cytological abnormalities only on follow-up visits as the medium used to collect baseline specimens was not suitable for cytological analysis. Subjects were classified as normal or low-grade squamous intraepithelial lesions (LSILs). No high-grade squamous intraepithelial lesions or invasive cervical cancer cases were diagnosed. Among women with a baseline HPV negative sample with valid follow-up cytological sample we evaluated incident cytological abnormalities according to type-specific HPV infections at follow-up (HPV 16 infected vs. non-infected, HPV 18 infected vs. non-infected, high-risk infected vs. non-infected, low-risk infected vs. non-infected, HPV 16-related types infected vs. non-infected, HPV 18-related vs. non-infected and infection with any HPV type vs. non-infected).

All tests of statistical hypotheses were made on the two-sided 5% level of significance with corresponding 95% confidence intervals. All analyses were made using the SAS software version 9.2 (SAS Corp). Procedure genmod was used to fit the Poisson regression.

## Results

### Characteristics of the cohort

**Figure 1:**
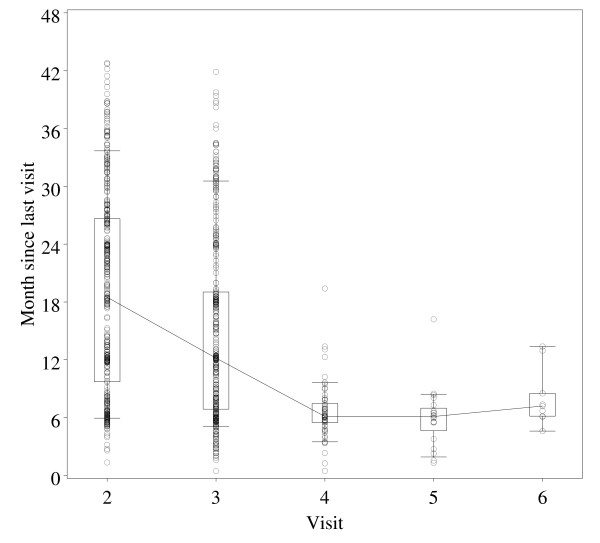
**Follow-up visits according to time (in months) since last visit**.

Of the 1,275 women enrolled in the study at baseline, only 380 (29.8%) women had at least one follow-up visit. Compared to women with adequate follow-up, the women with inadequate follow-up were slightly younger at study entry (>18 years 85.5% vs. 90.3%), less likely to have genital warts (7.6% vs. 8.2%), less likely to be pregnant at time of examination (11.8% vs. 15.0%) but more likely to be HIV positive (6.2 vs. 5.8%) and more likely to have initiated sex below 15 years (15.0% vs.10%). There was no difference at baseline between women who were adequately followed up and those who were not with respect to HPV positivity (74.6% vs. 74.7%) and the number of lifetime sexual partners (≥2 or more; 74.8% vs. 74.4%). The time between visits was highly variable. The median time to the 1^st ^follow-up visit was 18.5 months, with inter - quartile range of 9.7 to 26.6 months (Figure 1).

Table 1 shows HPV incidence and clearance between March 2003 and December 2006. The overall prevalence with any HPV type at baseline was 75.3% with cumulative positivity of 86.6%. Except for HPV 34, all the common high- and low-risk types were detected. The 5 most common high-risk types cumulatively detected in descending order were HPV 51 (n = 76, 20.0%), HPV 52 (n = 71, 18.7%), HPV 16 (n = 58, 15.3%), HPV 18 (n = 56, 14.7%) and HPV 33 (n = 52, 13.7%). Of the most common low-risk types, HPV 6 (n = 94, 24.7%), HPV 11 (n = 62, 16.3%) and HPV 66 (n = 35, 9.2%) were frequently detected. Cumulatively, high-risk HPV types (68.2%) were more frequently detected than low-risk types (55.0%). Single infections (55.3%) were slightly more common than multiple infections (47.6%) as were HPV 16-related types (48.6%) compared to HPV 18-related types (28.2%). Cumulatively, 75 (19.7%) infections were uncharacterized (HPV X) after the first round of testing and went through a second round of testing for an additional 17 types. Thirty-three (33) women could be assigned one or more specific types after the second round of testing leaving 42 (11.1%) women as HPV X. Further work on HPV X is currently ongoing which is detecting other types not identified in the established SPF-10.

**Table 1:** Cumulative positivity of HPV infections among 380 women up to 3 follow-up visits between March 2003 and December 2006 in Kampala, Uganda

	Prevalence at entry (%)	Cumulative positivity (%)	Positive once (%)	Positive ≥ 2 visits (%)
Infections with high-risk HPV types				

16	42 (11.1)	58 (15.3)	49 (12.9)	9 (2.4)
18	47 (12.4)	56 (14.7)	50 (13.2)	6 (1.6)
31	24 (6.3)	43 (11.3)	36 (9.5)	7 (1.8)
33	41 (10.8)	52 (13.7)	46 (12.1)	6 (1.6)
35	22 (5.8)	40 (10.5)	33 (8.7)	7 (1.8)
39	18 (4.7)	26 (6.8)	21 (5.5)	5 (1.3)
45	11 (2.9)	20 (5.3)	16 (4.2)	4 (1.1)
51	54 (14.2)	76 (20.0)	59 (15.5)	17 (4.5)
52	49 (12.9)	71 (18.7)	52 (14.2)	17 (4.5)
56	32 (8.4)	54 (14.2)	47 12.4)	7 (1.8)
58	8 (2.1)	22 (5.8)	19 (5.0)	3 (0.8)
59	8 (2.1)	15 (3.9)	12 (3.2)	3 (0.8)
68-73	20 (5.3)	35 (9.2)	30 (7.9)	5 (1.3)
39-68-73	8 (2.1)	11 (2.9)	11 (2.9)	0 (0.0)

Infections with low-risk HPV types				

6	74 (19.5)	94 (24.7)	82 (21.6)	12 (3.2)
11	55 (14.5)	62 (16.3)	59 (15.5)	3 (0.8)
34	0 (0.0)	0 (0.0)	0 (0.0)	0 (0.0)
40	17 (4.5)	20 (5.3)	17 (4.5)	3 (0.8)
42	2 (0.5)	2 (0.5)	2 (0.5)	0 (0.0)
43	19 (5.0)	22 (5.8)	19 (5.0)	3 (0.8)
44	6 (1.6)	14 (3.7)	8 (2.1)	6 (1.6)
53	12 (3.2)	17 (4.5)	15 (3.9)	2 (0.5)
54	9 (2.4)	21 (5.5)	15 (3.9)	6 (1.6)
66	20 (5.3)	35 (9.2)	27 (7.1)	8 (2.1)
70	11 (2.9)	18 (4.7)	13 (3.4)	5 (1.3)
74	2 (0.5)	7 (1.8)	4 (1.1)	3 (0.8)
HPV X^a^	26 (6.8)	75 (19.7)	66 (17.4)	9 (2.4)
HPV other X ^b^	8 (2.1)	42 (11.1)	38 (10.0)	4 (1.1)

Number of women with:				

- Any HPV types	286 (75.3)	329 (86.6)	122 (32.1)	207 (54.5)
- High-risk HPV types	197 (51.8)	259 (68.2)	139 (36.6)	120 (31.6)
- Low-risk HPV types	167 (43.9)	209 (55.0)	144 (37.9)	65 (17.1)
- Single infections	102 (26.8)	210 (55.3)	142 (37.4)	68 (17.9)
- Multiple infections	152 (40.0)	181 (47.6)	124 (32.6)	57 (15.0)
-HPV 16--related ^c^	127 (33.4)	178 (46.8)	113 (29.7)	65 (17.1)
-HPV 18--related ^d^	77 (20.3)	107 (28.6)	82 (21.6)	25 (6.6)

^a ^HPV X includes types 26, 30, 55, 61, 62, 64, 67, 69, 71, 82, 83, 84, 87, 89, 90 and other unknown types

^b^HPV-other X, i.e. type unknown

^c ^Includes HPV 16, 31, 33, 35, 52 and 58;

^d ^Includes HPV 18, 39, 45, 59, 68

### Incidence of HPV infections

Table 2 shows the type-specific HPV incidence rates of 155 HPV negative women at baseline followed up between March 2003 and December 2006. Sixty-nine women had an incident HPV infection during 226 person-years of observation, reflecting an incidence rate of 30.5 per 100 person-years. Type-specific incidence rates ranged between 0.8 and 4.2 per 100 person-years of observation for high-risk HPV types and between 1.1 and 3.4 per 100 person-years for common low-risk types. Incident high-risk types (20.9 per 100 person-years) were more frequent than low-risk HPV types (10.6 per 100 person-years). Incident infections with HPV 16-related were twice as likely as HPV 18-related types (10.8 and 5.6 per 100 person-years, respectively). Incident HPV infections were marginally associated with HIV positivity (RR = 2.8, 95% CI: 0.9 - 8.3) but not with age at study entry, and lifetime numbers of sexual partners (Table 3).

**Table 2:** HPV incidence and clearance between March 2003 and December 2006, Kampala, Uganda

	Incidence			Clearance		
	
HPV type	Incident cases^a^	Person years	Incidence rate per 100 p. yrs	Number cleared	Person years	Clearance rate per 100 p. yrs
High-risk types						

16	16	487	3.3	45	63	71.4
18	10	491	2.0	47	59	79.7
31	20	727	2.8	25	29	86.2
33	13	407	3.2	43	43	100.0
35	19	527	3.6	22	52	42.3
39	8	540	1.5	17	29	58.6
45	9	696	1.3	11	20	55.0
51	25	602	4.2	54	90	60.0
52	23	674	3.4	45	84	53.6
56	24	697	3.4	40	50	80.0
58	14	558	2.5	10	11	90.9
59	8	508	1.6	10	19	52.6
68	10	749	1.3	5	1	100.0
68-73	15	529	2.8	25	39	64.1
39-68-73	3	399	0.8	9	11	81.8

Low-risk types						

6	22	651	3.4	69	107	64.5
11	8	440	1.9	59	116	50.9
34	0	0	0.0	0	0	0.0
40	4	288	1.1	18	26	46.2
42	0	0	0.0	2	1	100.0
43	3	225	1.3	18	23	78.3
44	8	713	1.1	5	6	83.3
53	5	289	1.7	13	18	72.2
54	14	735	1.9	14	15	93.3
66	15	649	2.4	19	38	50.0
70	7	600	1.2	11	9	100.0
74	5	373	1.3	2	1	100.0
HPV X^b ^other types	50	701	7.1	39	55	70.9
HPV X ^c^	33	550	6.0	15	18	83.3

Number of women with:						

- Any HPV	69	226	30.5	143	459	31.2
- High-risk	85	407	20.9	145	344	42.2
- Low-risk	49	461	10.6	138	292	47.3
- Single infections	115	532	21.6	104	218	47.7
- Multiple infections	42	508	8.3	133	241	55.2
- HPV 16-related ^d^	59	547	10.8	106	208	51.0
- HPV 18-related ^e^	36	640	5.6	76	116	65.5

Note; The figures do not add up because of missing figures

Abbreviations: P. yrs, person-years; HR, high-risk; LR, low-risk

^a ^Crude numbers

^b ^HPV types detected at a 2^nd ^round of testing: HPV 26, 30, 55, 61, 62, 64, 67, 69, 71, 82, 83, 84, 87, 89, 90, and other unknown types

^c^HPV X, i.e. type unknown

^d ^Includes HPV 16, 31, 33, 35, 52 and 58

^e ^Includes HPV 18, 39, 45, 59, 68

**Table 3:** HPV incident and clearance cases and rates per 100 person-years and RR according to different women's characteristics

Baseline Characteristics	Incidence				Clearance			
	
	Distribution women (%)	Person years	Rate per 100 p. yrs	RR (95% CI)^a^	Distribution women (%)	Person years	Rate per 100 p. yrs	RR (95% CI)^a^
Age (yrs) at study entry								

21 -- 24	78 (50.3)	115	25.3	1 (ref.)	145 (47.4)	72	9.8	1 (ref.)
18 -- 20	62 (40.0)	92	32.6	1.2 (0.7 -- 2.0)	128 (41.8)	64	7.8	0.7 (0.5-1.0)
12 -- 17	15 (9.7)	19	53.3	2.2 (1.0 -- 4.5)	33 (10.8)	16	0	0.9 (0.5-1.5)

Lifetime number of sexual partners								

1	47 (30.3)	66	30.1	1 (ref.)	73 (23.9)	36	8.3	1 (ref.)
2	45 (29.0)	78	26.8	0.8 (0.4 -- 1.5)	91 (29.7)	45	11.1	0.9 (0.6-1.5)
3	36 (23.2)	46	36.8	1.5 (0.8 -- 2.9)	73 (23.9)	36	2.7	0.9 (0.5-1.4)
4 or more	27 (17.4)	34	32	1.1 (0.5 -- 2.3)	69 (22.5)	34	8.7	1.0 (0.6-1.6)

HIV status								

Negative	148 (95.5)	212	29.7	1 (ref.)	272 (88.9)	135	8.9	1 (ref.)
Positive	4 (2.6)	6	65	2.8 (0.9 -- 8.3)	20 (6.5)	10	0	0.3 (0.1-0.8)
Unknown	3 (1.9)	7	27.2	-	14 (4.6)	7	0	-

Multiplicity of HPV types ^b^								

No infection	--	--	--	--	28 (9.2)	14	14.5	1 (ref.)
Single	--	--	--	--	108 (35.3)	54	5.6	0.5 (0.3-1.0)
2 or more types	--	--	--	--	170 (55.6)	841	8.3	0.6 (0.4-1.2)

Note 1: The figures do not add up because of missing values

Note 2: The denominator is person-years

Abbreviations: CI, Confidence Intervals; p. yrs, person-years

^a ^adjusted for age, number of sexual partners and HIV infection

^b ^2 or more types compared to 1 infection: for clearance 1.3 (0.9-1.8)

### Clearance of HPV infections

Overall, 143 women with prevalent infections cleared their infections during 459 person-years of observation reflecting complete clearance for all HPV types of 31.2% (Table 2). Clearance for specific types ranged between 42.3% and 100.0% for high-risk and 50% and 100.0% for low-risk HPV types. Women with high-risk types (42.2%) cleared their HPV infections as much as those with low-risk types (47.3%). However, women with HPV 16-related types (51.0%) cleared their infections less than those with HPV 18-related types (65.5%). Clearance was only associated with HIV seronegativity (RR = 0.3, 95% CI: 0.1 - 0.8) but not with age at study entry, lifetime number of sexual partners, and multiplicity of infections.

Incident HPV infections were common in both HIV positive (rates between 2.2 and 15.4 per 100 person-years) and HIV negative women (rates between 0.8 and 4.2 per 100 person-years) [See Additional file 1: Table S1 for original data used to perform this analysis]. Compared to HIV negative women, the risk for incident infections was elevated for low-risk HPV types (RR = 3.3, 95% CI: 1.4 - 7.8) and HPV 16-related types (RR = 3.0, 95% CI: 1.3 - 6.9) in HIV positive women and the difference was statistically significant. The risk for any HPV type (RR = 2.8, 95% CI: 0.9 - 8.3) and for high-risk types (RR = 2.1, 95% CI: 0.9 - 5.1) was of borderline statistical significance among HIV positive compared to HIV negative women. There was no difference in risk for HPV 18-related types (RR = 1.8, 95% CI: 0.5 - 6.2); single infections (RR = 1.1, 95% CI: 0.5 - 2.3), multiple infections (RR = 2.2, 95% CI: 0.6 - 7.6) and HPV - other X (RR = 0.9, 95% CI: 0.5 - 1.8) among HIV positive compared to HIV negative women. Clearance was significantly less for any HPV and multiple infections among HIV positive women compared to HIV negative women (Adjusted clearance = 0.2, 95% CI: 0.1 - 0.7) and the difference was statistically significant. There was no statistically significant difference in clearance of any high- or low-risk HPV types, HPV 16-related, HPV 18-related and single infections between HIV positive and negative women.

### Prevalence and Incidence of LSIL

Three hundred seventy-eight (378) of 380 (99.5%) women with at least one follow-up visit provided a sample for liquid-based cytology. Twelve (12) samples were inadequate leaving 365 samples for cytological analysis; of these 365 samples, 53 (14.5%) had LSILs. None of the women had HSIL or cancer. One hundred and seventy-three (173) women provided at least 2 samples for cytologic analysis of which, 5 and 3 samples were inadequate at 1^st ^and 2^nd ^follow-up visit leaving 168 and 170 samples, respectively, available for cytological analysis. The prevalence of LSILs at 1^st ^cytologic sample was 15/168 (8.9%) and declined to 9/170 (5.3%) at the 2^nd ^visit. Evaluation incident LSIL was possible for 150/155 (96.8%) women who were HPV DNA negative at baseline. Twenty-two (22) of 150 (14.7%) developed LSILs between baseline and 1^st ^follow-up (Table 4). Incident LSILs appear to be more frequent among women infected with HPV 18-related types compared to women not infected with those types (RR = 3.5, 95% CI: 1.0 - 11.8), though the difference is of borderline statistical significance.

**Table 4:** LSIL incidence among women with normal cytology followed upbetween March 2003 and December 2006 in Kampala, Uganda

HPV type	No. of women	Person years	Incident LSIL	Incidence rate per 100 p. yrs	Crude RR (95% CI)^a^ Infected/non-infected	p-value
HPV 16						

Non-infected	142	114	21	18.4	1	
Infected	8	9	1	11.1	0.5 (0.1 -- 3.9)	0.52

HPV 18						

Non-infected	147	120	21	17.5	1	
Infected	3	3	1	33.3	2.2 (0.3 -- 16.2)	0.45

High-risk HPV types						

Non-infected	104	81	12	14.8	1	
Infected	46	43	10	23.3	1.4 (0.6 -- 3.4)	0.39

Low-risk HPV types						

Non-infected	122	99	20	20.2	1	
Infected	28	24	2	8.3	0.4 (0.1 -- 1.8)	0.25

HPV 16-related ^b^						

Non-infected	124	98	18	18.4	1	
Infected	26	25	4	16	0.8 (0.3 -- 2.3)	0.64

HPV 18-related ^c^						

Non-infected	142	117	19	16.2	1	
Infected	8	64	3	50	3.5 (1.0 -- 11.8)	0.05

Any HPV type						

Non-infected	76	58	10	17.2	1	
Infected	74	65	12	18.5	1.0 (0.4 -- 2.5)	0.91

Abbreviations: CI, confidence intervals; p. yrs, person--years

^a^Further adjusted for age, HIV, lifetime number of sexual partners did not change the results

^b^Includes HPV 16, 31, 33, 35, 52 and 58

^c^Includes HPV 18, 39, 45, 59 and 68

### HIV testing at follow-up

The majority of women who were tested HIV positive during follow-up had declined HIV testing at enrolment; We suppose that the women already knew they were HIV positive therefore we chosen to not provide the analysis of HIV incidence during follow-up.

## Discussion

The observed high incidence rate of HPV infections among the young women in our study is similar to finding described in previous studies [15-17]. A woman's risk for incident infection was defined by her HIV serostatus. Indeed, women infected with HIV were twice as likely as HIV negative women to have incident infection with high- or low-risk HPV types in agreement with published studies [18]. The difference in risk for incident HPV 16 and HPV18 infections was not statistically significant in both HIV positive and negative women. Safaeian *et al*. (2008) [9] in a population-based study in a rural district of Uganda, found a 3-fold elevated risk for HPV 16 and HPV 18 among HIV positive compared to HIV negative women. In their study, the study population consisted of both young and old women aged 15 - 55 years and this could explain the difference between our findings and theirs. However, the limited sample size in our study could have limited our ability to detect a difference. Despite the limited age range of the women in our study, we found higher incidence rate for high- than for low-risk HPV types, similar to studies with a wide age ranges [19-21].

Our findings differ from studies conducted among adult populations who found that the 12-month clearance for low-risk HPV types was more than for high-risk types but no clear difference between infections with high-risk types other than HPV 16 [19]. The different sensitivity of HPV DNA detection assays used in other studies compared to ours and the age structure of the population of women studied might explain the observed differences. Nevertheless, HPV 16 tend to persist longer than other HPV types as observed in several follow-up studies of adult populations unlike our study population that comprised of young women whose HPV infections were more likely to be transient [22-24]. Clearance was associated with HIV seronegativity but not with age at study entry, lifetime number of sexual partners, and multiplicity of infections at the beginning of follow-up. The women's characteristics associated with clearance of infections with HPV are not consistent across studies [9,19,25,26]. For instance, some studies have reported less clearance of HPV infection in women with more lifetime sexual partners, a finding suggestive of past exposure or reactivated infections acquired earlier, which may not be prone to clearance. In our study, however, HPV infections were not associated with number of lifetime sexual partners perhaps because prior exposure to HPV infection may have been limited given the young age of our study population. Only 31.2% of women had cleared all their infections by the end of the follow-up period. Considering the median time to first follow-up in our study was 18.5 months, this period was probably long enough for a woman to clear an infection and get re-infected with the a different or even the same HPV type. Thus, it is possible that our observed clearance was likely to be of incident infections acquired subsequently rather than the initial prevalent infections that the woman had at baseline.

Despite the high incidence of HPV infections, the incidence of LSIL was relatively low (14.5%) which suggests that many young women who were infected with HPV had no cellular changes. Moscicki *et al*. (2001) [27], in a study of 496 adolescents and young women found that only 25% of those who acquired HPV infections developed LSILs. It is believed that LSIL is merely a manifestation of HPV infection [27] and indeed LSIL seemed to be more frequent among women infected with HPV 18-related types than those not infected with those types. The decline in prevalent LSIL from 8.9% to 5.3% between the 1^st ^and 2^nd ^visit in women who provided at least 2 adequate samples for cytologic analysis underscores the transient nature of HPV infection and associated cervical lesions among young women. Our findings therefore, imply that initiating screening for cervical cancer in young women is likely to detect transient low-grade cervical abnormalities. Current knowledge of the natural history of HPV infection and its associated cervical lesions suggest that conservative management, such as follow-up without aggressive surgical treatment may be indicated in young women with LSIL. Secondly, screening with HPV testing among young women is not useful as many women are likely to be HPV positive as shown by the cumulative HPV results.

Our study has strengths and limitations. We used a highly sensitive HPV testing PCR assay for HPV detection to show a high rate of acquisition of new HPV infections in a population of young women in an area with high-risk of cervical cancer. The same PCR assay was used for HPV detection at baseline. Given the high sensitivity of the PCR assay, under detection of HPV infections was unlikely in our study. Thus, new infections were unlikely to be infections previously missed and cleared infections were unlikely to be due to errors in follow-up samples.

Our study underscores the challenges of conducting follow-up studies in low resource countries where residence has no proper street address. Hence our study had significant loss to follow-up (70.2%) which could have introduced selection bias. Women were lost to follow-up because they could not be located, declined to have further specimens collected or were back in school at the time of scheduled visits. However, there was no difference between the women adequately followed up and those who were not regarding lifetime number of sexual partners, which is a strong and consistent risk factor for acquisition of HPV infections. Secondly, loss to follow-up was unlikely to be associated with the woman's HPV status because the woman was blinded of her HPV status throughout the follow-up period. We used prevalent rather than incident infections to assess clearance and as a result, some women could have lost an HPV infection between visits and this could have led to the underestimation of the clearance.

## Conclusions

In conclusion, we investigated incidence, clearance and potential risk factors of HPV infections among 380 young women followed up in Kampala, Uganda. Our study shows that incident HPV infections were frequently underscoring the importance of vaccination of young women before they become sexually active.

## Competing interests

The authors declare that they have no competing interests.

## Authors' contributions

CB & EW conceived the study. CB wrote the study protocols, coordinated the field work, designed data collection forms, including the questionnaires, clinical databases and wrote the manuscript. EW supervised CB throughout the study from its conception, preparation of study protocols, data and specimen collection, interpretation of the results and made substantial contributions to the manuscript. FWM & EKM supervised CB during fieldwork and made substantial comments to the manuscript. SS made the statistical analyses, produced the working and final tables, and wrote part of the methods section. BK, LJD, & WQ performed HPV detection and genotyping of the study samples, created the HPV database, wrote parts of the methods section and made substantial contributions to the manuscript. All authors read and approved the final manuscript.

## Supplementary Material

Additional file 1 **Table S1 - HPV incidence and clearance among HIV positive and HIV negative women, Kampala, Uganda**. original data used to perform analysis of clearance by HPV type. Click here for file
