# Supplementary material for: Type-specific incidence, clearance and predictors of cervical human papillomavirus infections (HPV) among young women: a prospective study in Uganda
Source: Infect Agent Cancer. 2010 Apr 9;5:7. doi: 10.1186/1750-9378-5-7 (PMC2873244; doi:10.1186/1750-9378-5-7)
Supplement: Additional file 1 — Table S1 - HPV incidence and clearance among HIV positive and HIV negative women, Kampala, Uganda. original data used to perform analysis of clearance by HPV type. [file 1750-9378-5-7-S1.DOC]

**Table 1 - HPV incidence and clearance among HIV positive and HIV negative women, Kampala, Uganda**

|  |  | Incidence | | |  |  |  |  | Clearance | |  |  |
| --- | --- | --- | --- | --- | --- | --- | --- | --- | --- | --- | --- | --- |
|  |  |  |  |  |  |  |  |  |  |  |  |  |
|  | HIV Negative | | HIV Positive | | HIV Positive/HIV Negative | | HIV Negative | | HIV Positive | | HIV Positive/HIV Negative |  |
|  |  |  |  |  |  |  |  |  |  |  |  |  |
| HPV type | Incident | Rate | Incident | Rate | Adjusted Incidence ^b^ |  | Number | Clearance | Number | Clearance | Adjusted clearance ^b^ |  |
|  | cases ^a^ | 100 p.yrs | cases ^a^ | 100 p.yrs | RR (95% CI) | p value | Cleared | Rate 100 p.yrs | Cleared | Rate 100 p.yrs | RR (95% CI) | p value |
| HR types |  |  |  |  |  |  |  |  |  |  |  |  |
| 16 | 13 | 3.0 | 2 | 6.7 | 2.7 (0.6 – 12.6) | 0.207 | 41 | 70.7 | 3 | 60.0 | 0.6 (0.1 – 2.5) | 0.484 |
| 18 | 9 | 2.1 | 1 | 3.0 | 1.8 (0.2 – 16.1) | 0.582 | 46 | 78.0 | 0 | 0.0 | 0 |  |
| 31 | 13 | 2.0 | 2 | 4.7 | 2.4 (0.5 – 11.8) | 0.269 | 21 | 80.8 | 2 | 66.7 | 1.0 (0.2 – 5.2) | 0.987 |
| 33 | 8 | 2.0 | 4 | 15.4 | 8.0 (2.1 – 30.6) | 0.002 | 38 | 100.0 | 5 | 83.3 | 0.6 (0.2 –1.7) | 0.360 |
| 35 | 14 | 2.9 | 3 | 9.1 | 6.4 (1.5 – 27.2) | 0.012 | 21 | 56.8 | 0 | 0.0 | 0 |  |
| 39 | 7 | 2.1 | 0 | 0.0 | 0 |  | 14 | 60.9 | 2 | 66.7 | 4.2 (0.2 – 84.7) | 0.343 |
| 45 | 8 | 1.3 | 0 | 0.0 | 0 |  | 8 | 66.7 | 1 | 14.3 | - |  |
| 51 | 23 | 4.2 | 1 | 3.7 | 0.9 (0.1 – 7.4) | 0.961 | 45 | 69.2 | 5 | 33.3 | 0.3 (0.1 – 0.8) | 0.024 |
| 52 | 21 | 3.4 | 1 | 2.8 | 0.6 (0.1 – 4.9) | 0.661 | 36 | 55.4 | 5 | 55.6 | 1.1 (0.4 – 3.2) | 0.886 |
| 56 | 21 | 3.3 | 1 | 2.6 | 0.9 (0.1 – 7.0) | 0.932 | 34 | 89.5 | 2 | 28.6 | 0.1 (0.0 – 0.9) | 0.044 |
| 58 | 11 | 2.2 | 3 | 8.6 | 4.4 (1.1 – 17.3) | 0.036 | 9 | 90.0 | 0 | 0.0 | 0 |  |
| 59 | 6 | 1.3 | 1 | 3.3 | 3.1 (0.3 – 28.9) | 0.330 | 8 | 50.0 | 2 | 66.7 | - |  |
| 68 | 8 | 1.2 | 1 | 2.2 | 1.5 (0.2 – 12.8) | 0.695 | 4 | 100.0 | 1 | 25.0 | - |  |
| 68-73 | 9 | 1.9 | 3 | 10.7 | 10.1 (2.4 – 42.4) | 0.002 | 17 | 60.7 | 5 | 62.5 | 2.1 (0.6- 6.9) | 0.228 |
| 39-68-73 | 3 | 0.8 | 0 | 0.0 | 0 |  | 7 | 70.0 | 1 | 100.0 | - |  |
|  |  |  |  |  |  |  |  |  |  |  |  |  |
| LR types |  |  |  |  |  |  |  |  |  |  |  |  |
| 6 | 15 | 2.6 | 4 | 10.3 | 5.1 (1.6 – 16.7) | 0.007 | 65 | 67.7 | 3 | 42.9 | 0.8 (0.2 – 2.7) | 0.660 |
| 11 | 7 | 1.7 | 1 | 4.4 | 4.7 (0.5 – 47.7) | 0.193 | 49 | 50.5 | 6 | 50.0 | 1.1 (0.4 – 3.1) | 0.846 |
| 34 | 0 | 0.0 | 0 | 0.0 | 0 |  | 0 | 0.0 | 0 | 0.0 | 0 |  |
| 40 | 3 | 1.1 | 0 | 0.0 | 0 |  | 15 | 79.0 | 1 | 50.0 | 0.2 (0.0 – 2.5) | 0.210 |
| 42 | 0 | 0.0 | 0 | 0.0 | 0 |  | 1 | 100.0 | 0 | 0.0 | 0 |  |
| 43 | 3 | 1.4 | 0 | 0.0 | 0 |  | 17 | 94.4 | 1 | 20.0 | 0.1 (0.0 – 0.7) | 0.028 |
| 44 | 6 | 1.2 | 1 | 2.9 | 2.8 (0.3 – 26.0) | 0.357 | 5 | 100.0 | 0 | 0.0 | 0 |  |
| 53 | 4 | 1.5 | 0 | 0.0 | 0 |  | 13 | 72.2 | 0 | 0.0 | 0 |  |
| 54 | 12 | 1.8 | 1 | 2.2 | 1.1 (0.1 – 8.6) | 0.953 | 10 | 76.9 | 0 | 0.0 | 0 |  |
| 66 | 12 | 1.9 | 2 | 4.9 | 2.2 (0.5 - 10.7) | 0.315 | 18 | 54.6 | 1 | 25.0 | 0.4 (0.0 –3.8) | 0.425 |
| 70 | 6 | 1.0 | 1 | 2.5 | 3.3 (0.4 – 30.9) | 0.293 | 11 | 100.0 | 0 | 0.0 | 0 |  |
| 74 | 5 | 1.5 | 0 |  |  |  |  |  |  |  |  |  |
| Number of women with: | |  |  |  |  |  |  |  |  |  |  |  |
| - Any HPV | 63 | 29.7 | 4 | 66.7 | 2.8 (0.9 – 8.3) | 0.007 | 138 | 29.7 | 3 | 7.5 | 0.2 (0.1 – 0.7) | 0.011 |
| - Any High-risk | 78 | 20.3 | 6 | 40.0 | 2.1 (0.9 – 5.1) | 0.088 | 135 | 46.4 | 6 | 20 | 0.5 (0.2 – 1.0) | 0.064 |
| - Any Low-risk | 39 | 9.3 | 7 | 29.2 | 3.3 (1.4 – 7.8) | 0.005 | 131 | 50.8 | 0.5 | 22.7 | 0.4 (0.2 – 1.1) | 0.065 |
| HPV-other X ^c^ | 47 | 7.4 | 2 | 4.6 | 0.9 (0.5 - 1.8) | 0.515 | 38 | 73.0 | 1 | 33.3 | 0.5 (0.1 - 4.4) | 0.567 |
| - HPV X ^d^ | 32 | 6.4 | 0 | 0.0 | 0 |  | 15 | 78.9 | - | - | - |  |
| - HPV 16 related ^e^ | 49 | 9.7 | 7 | 24.1 | 3.0 (1.3 –6.9) | 0.011 | 96 | 55.5 | 4 | 23.5 | 0.4 (0.2 – 1.2) | 0.109 |
| - HPV 18 related ^f^ | 31 | 5.4 | 3 | 8.1 | 1.8 (0.5 – 6.2) | 0.360 | 69 | 67.0 | 4 | 44.4 | 0.6 (0.2 – 1.8) | 0.419 |
| - Single infection | 103 | 21.8 | 7 | 25.0 | 1.1 (0.5 – 2.3) | 0.888 | 95 | 46.8 | 4 | 40 | 1.1 (0.4 – 3.1) | 0.851 |
| - Multiple infections | 36 | 7.6 | 3 | 15.0 | 2.2 (0.6 – 7.6) | 0.209 | 123 | 62.1 | 5 | 19.2 | 0.3 (0.1 – 0.7) | 0.006 |
|  |  |  |  |  |  |  |  |  |  |  |  |  |

Note; The figures do not add up because of missing figures

Abbreviations: CI, Confidence Intervals; p.yrs, person - years; HR, High risk; LR, Low risk

^a^ Crude numbers

^b^ Adjusted for age and lifetime sexual partners

^c^ HPV-other X, i.e. type unknown

^d^ HPV types 26, 30, 55, 61, 62, 64, 67, 69, 71, 82, 83, 84, 87, 89, and 90 and other unknown types

^e^ Includes HPV 16, 31, 33, 35, 52 and 58

^f^ Includes HPV 18, 39, 45, 59 and 68
